# Evidence for treatment with estradiol for women with SARS-CoV-2 infection

**DOI:** 10.1186/s12916-020-01851-z

**Published:** 2020-11-25

**Authors:** Ute Seeland, Flaminia Coluzzi, Maurizio Simmaco, Cameron Mura, Philip E. Bourne, Max Heiland, Robert Preissner, Saskia Preissner

**Affiliations:** 1 Institute of Physiology and Science-IT, Charité – Universitätsmedizin Berlin, corporate member of Freie Universität Berlin, Humboldt-Universität zu Berlin, and Berlin Institute of Health, Philippstrasse 12, 10115 Berlin, Germany; 2 Department Medical and Surgical Sciences and Biotechnologies, Sapienza University of Rome, 04100 Latina, Italy; 3 Unit of Anesthesia and Intensive Care Medicine, Sant’Andrea University Hospital, 00189 Rome, Italy; 4 Department Neurosciences, Mental Health and Sensory Organs, Sapienza University of Rome, 00189 Rome, Italy; 5 Advanced Molecular Diagnostic Unit, Sant’Andrea University Hospital, 00189 Rome, Italy; 6 School of Data Science and Department of Biomedical Engineering, University of Virginia, Charlottesville, VA USA; 7 Department Oral and Maxillofacial Surgery, Charité – Universitätsmedizin Berlin, corporate member of Freie Universität Berlin, Humboldt-Universität zu Berlin, and Berlin Institute of Health Augustenburger Platz 1, 13353 Berlin, Germany

**Keywords:** Sex, Women, SARS-CoV-2, COVID-19, Estradiol, Hormone treatment, ACE2

## Abstract

**Background:**

Given that an individual’s age and gender are strongly predictive of coronavirus disease 2019 (COVID-19) outcomes, do such factors imply anything about preferable therapeutic options?

**Methods:**

An analysis of electronic health records for a large (68,466-case), international COVID-19 cohort, in 5-year age strata, revealed age-dependent sex differences. In particular, we surveyed the effects of systemic hormone administration in women. The primary outcome for estradiol therapy was death. Odds ratios (ORs) and Kaplan-Meier survival curves were analyzed for 37,086 COVID-19 women in two age groups: pre- (15–49 years) and peri-/post-menopausal (> 50 years).

**Results:**

The incidence of severe acute respiratory syndrome coronavirus 2 (SARS-CoV-2) infection is higher in women than men (by about + 15%) and, in contrast, the fatality rate is higher in men (about + 50%). Interestingly, the relationships between these quantities are linked to age: pre-adolescent girls and boys had the same risk of infection and fatality rate, while adult premenopausal women had a significantly higher risk of infection than men in the same 5-year age stratum (about 16,000 vs. 12,000 cases). This ratio changed again in peri- and postmenopausal women, with infection susceptibility converging with men. While fatality rates increased continuously with age for both sexes, at 50 years, there was a steeper increase for men. Thus far, these types of intricacies have been largely neglected. Because the hormone 17ß-estradiol influences expression of the human angiotensin-converting enzyme 2 (ACE2) protein, which plays a role in SARS-CoV-2 cellular entry, propensity score matching was performed for the women’s sub-cohort, comparing users vs. non-users of estradiol. This retrospective study of hormone therapy in female COVID-19 patients shows that the fatality risk for women > 50 years receiving estradiol therapy (user group) is reduced by more than 50%; the OR was 0.33, 95% CI [0.18, 0.62] and the hazard ratio (HR) was 0.29, 95% CI [0.11,0.76]. For younger, pre-menopausal women (15–49 years), the risk of COVID-19 fatality is the same irrespective of estradiol treatment, probably because of higher endogenous estradiol levels.

**Conclusions:**

As of this writing, still no effective drug treatment is available for COVID-19; since estradiol shows such a strong improvement regarding fatality in COVID-19, we suggest prospective studies on the potentially more broadly protective roles of this naturally occurring hormone.

**Supplementary Information:**

The online version contains supplementary material available at 10.1186/s12916-020-01851-z.

## Background

### Epidemiological data

Early epidemiological observations indicated that severe acute respiratory syndrome coronavirus 2 (SARS-CoV-2) infects all age groups, but with a higher rate among men (58.1%) than women (41.9%) [1]. The fraction of males among critically ill patients testing positive for coronavirus disease 2019 (COVID-19) is higher, and the outcome is far worse than for women (among casualties, 66% are male and 34% are female) [2]. From a medical perspective of sex and gender, considering the epidemiological data in a more differentiated manner—based, for instance, on age, sex, and lifestyle/behavior patterns—may enable the identification of sub-populations with increased susceptibility (or resistance) to SARS-CoV-2 infection.

As an example of such nuances, consider that spread of the viral infection exhibits a sex-dependence, and also a higher fatality rate among older patients, > 60 years in age [3, 4]. In addition, smoking behavior has been one of the most appreciated gender differences thus far, and it may underlie the far worse outcomes in men than in women [5]; however, this trend has not been confirmed beyond a handful of countries, and the causal aspects of these trends are likely to be rather complex.

### Pathophysiological context

SARS-CoV-2 virions use the angiotensin-converting enzyme 2 (ACE2) as a host-cell receptor [6] for viral uptake. Human ACE2 is an essential part of the renin-angiotensin system (RAS) and is encoded on the X chromosome [7]. ACE2 is widely distributed in tissues, including lung alveolar (type II) epithelial cells, the vascular endothelium, heart, kidney, and testis [8]. It has extensive vascular and organ-protective functions mediated via angiotensin (Ang 1-7), by the angiotensin II receptor type 2 (AT2), and the Mas receptor (MasR). Estrogens downregulate the angiotensin II receptor type 1 (AT1R) signaling pathway and inhibit angiotensin-converting enzyme (ACE) activity [9, 10]. This classical ACE/AngII/AT1R regulatory axis counter-regulates—upregulates—the ACE2/Ang 1-7/MasR axis in the case of high estrogen levels [11]. 17ß-estradiol increases ACE2 activity in the adipose tissue, kidneys, and myocardium [12, 13].

Based on specific differences in genetics and sex hormone levels, the lower prevalence of SARS-CoV-2 infection in older women and men compared to younger people could be triggered by (i) conditions that are associated with a decrease in ACE2 expression such as older age [14] or (ii) decreased estradiol levels in postmenopausal women. Moreover, the regulation of immune cells and cytokine activity are also linked to sex hormone levels. Estradiol has beneficial effects on immune cell regulation by inhibiting interleukin 6 (IL6) and stabilizing the immune system in women. In terms of disease etiology, this could represent another set of factors that underlies the sex differences in pulmonary and vascular symptoms, severity, and outcomes of COVID-19.

## Methods

### Objectives of this study

As is known from the literature, endogenous estradiol has beneficial cardiovascular and immune system-stabilizing effects in premenopausal women, as well as within pregnancy [15, 16]. We hypothesize that exogenous estradiol intake is a protective strategy in pre- and post-menopausal women suffering from SARS-CoV-2 infection.

This study assesses the effects of exogenous sex hormone intake from oral contraceptives by premenopausal women and estradiol hormone therapy by postmenopausal women with SARS-CoV-2 infection or COVID-19 disease on the outcome death. We focused on estradiol because 82% of women over age 50 who are administered exogenous hormones are prescribed estradiol; only 22% of these women take additional progestins.

### Database and inclusion criteria

COVID-19 patients were identified via the ICD-10 code U07.1 or the presence of a SARS-CoV-2-related RNA diagnosis within the last 7 months (for details, see Additional file 1). Hormone use was identified via RxNorm codes 4083 (estradiol), 4124 (ethinyl estradiol), progestins VA:HS800, and systemic contraceptives VA:HS200. The data were collected from electronic health records (EHRs) in a TriNetX Real-World database provided by a global health research network, with healthcare organizations spanning 17 countries. This system deploys a linked and continually updated global health research network representing over 300 million patients.

In the present work, we conducted a retrospective analysis of a large international COVID-19 cohort comprising 68,466 cases. Sub-cohorts were disaggregated by age (15+ years) and sex before statistical analyses were performed. The incidence data are presented in age steps of 5 years, from 5 to 86+ years for both sexes. Because of the expected estradiol hormone status, two subgroups of the female cohort were analyzed: we defined 15–49 years as the younger (pre-menopausal) group and 50+ for the older (post-menopausal) group. The group comparison was calculated for COVID-19–positive patients with supplemental estradiol versus subjects without estradiol in the respective age strata.

### Real-world evidence data

Using real-world evidence (RWE) data affords several advantages over more traditional approaches. Prospective, placebo-controlled, randomized, double-blinded multicenter clinical trials are the gold standard of evidence generation in medicine; however, such efforts are often slow and expensive. Furthermore, classical trials generally represent artificial situations with well-selected—and thus non-representative—pools of patients (e.g., often young men with few to no co-morbidities). Some of these issues can be alleviated by turning to RWE gleaned from EHR-derived data. Despite this, one disadvantage of retrospective, implicit recruitments is the missing randomized placebo arm of the statistical study. Thus, generating suitable control cohorts was an important aspect of our RWE-based study. One significant advantage of RWE is the large number of patients who can be recruited—this, in turn, yields relatively tighter confidence intervals and allows for cohort balancing or patient matching. In non-RWE studies, a confounder analysis can be performed, but doing so is made more difficult because of the relatively small number of patients enrolled.

### TriNetX

The TriNetX platform ensures data quality control by processes and procedures triggered in response to questions about the data provided. Datasets do not leave hospitals: queries are executed in a federated manner, and only aggregated results are visible on TriNetX. TriNetX provides details on the data provenance and/or offers the necessary access to audit the processes. TriNetX also makes the data available for third-party audit.

### Statistical analysis

TriNetX analytics tools were used to obtain baseline characteristics, balance cohorts with propensity score matching, and analyze outcomes of interest in the final cohorts. The index event for each analysis was selected as the diagnosis of SARS-Cov2 infection within the last 7 months (Additional file 1). Baseline characteristics, including demographics, diagnoses, procedures, and medication, were obtained. Propensity score matching was used to balance cohorts. Propensity scores matched cohorts 1:1 using a nearest neighbor greedy matching algorithm with a caliper of 0.25 times the standard deviation. The primary outcome was defined as death. Measures of association, including risk differences, risk ratios, and odds ratios, with their respective 95% CIs, were calculated. In addition, Kaplan-Meier curves were generated for each analysis.

## Results

**Fig. 1:**
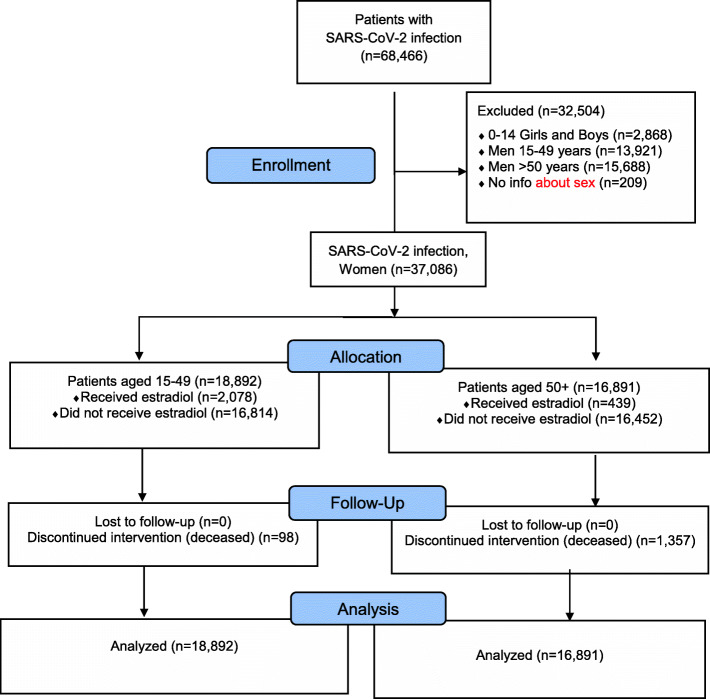
Our CONSORT flow diagram; major stages are indicated in the blue boxes

The consolidated standard of reporting trial (CONSORT) flow diagram, shown in Fig. 1, illustrates the data extraction process from the TriNetX Real-World database. The full cohort size of *n* = 68,466 patients consists of 37,086 women and 29,609 men who are older than 15 years (database accessed on July 16, 2020) and who are SARS-CoV-2 infected. After excluding (i) boys and girls up to 14 years of age, (ii) all men, and (iii) those without gender information, a sub-cohort of 37,086 women was used for further analysis of sex hormone status.

**Fig. 2:**
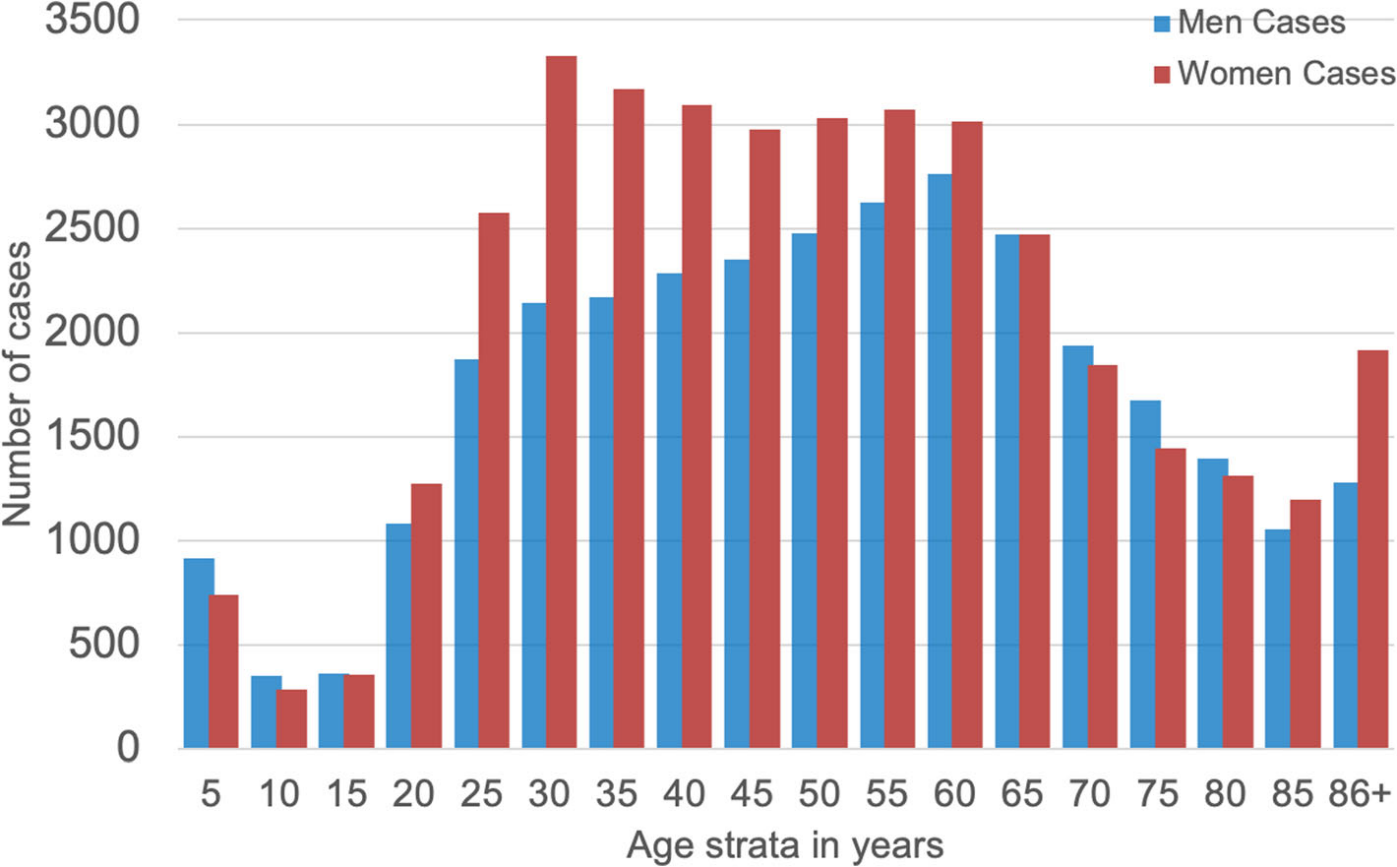
Absolute cases of COVID-19 in our cohort, disaggregated by women (red) and men (blue)

Regarding sex and gender, analyzing the data in terms of 5-year age strata, the incidence rates of women and men with known SARS-CoV-2 infection can be seen to significantly differ by sex (Fig. 2) (*t* test, *p* < 0.01). The highest SARS-CoV-2 infection numbers among women, compared to age-matched men, is observed in women with what would be expected to be high levels of serum estradiol—i.e., between 15 and 49 years of age. The incidence of COVID-19 in women in this pre-menopausal age group is about + 15% higher versus age-matched men (*n* = 21,229 women vs. *n* = 15,918 men). Starting with 60 and up to 85 years of age, the data show a relative decrease in frequency among women (*n* = 7334 women vs. *n* = 7171 men). In older women and men (age strata 65 to 80), the statistical susceptibility to viral infection converges (see the closely-matched red and blue pairs of bars in Fig. 2, in the range of 65–85 years old). Near the end of life, defined as 85+ years old, the incidence of SARS-CoV-2 infection is higher in women (*n* = 5865) than in men (*n* = 5406). At the opposite end of the age spectrum, all pre-adolescents (girls and boys) have similar risks of infection.

### Death rate for the full cohort

**Fig. 3:**
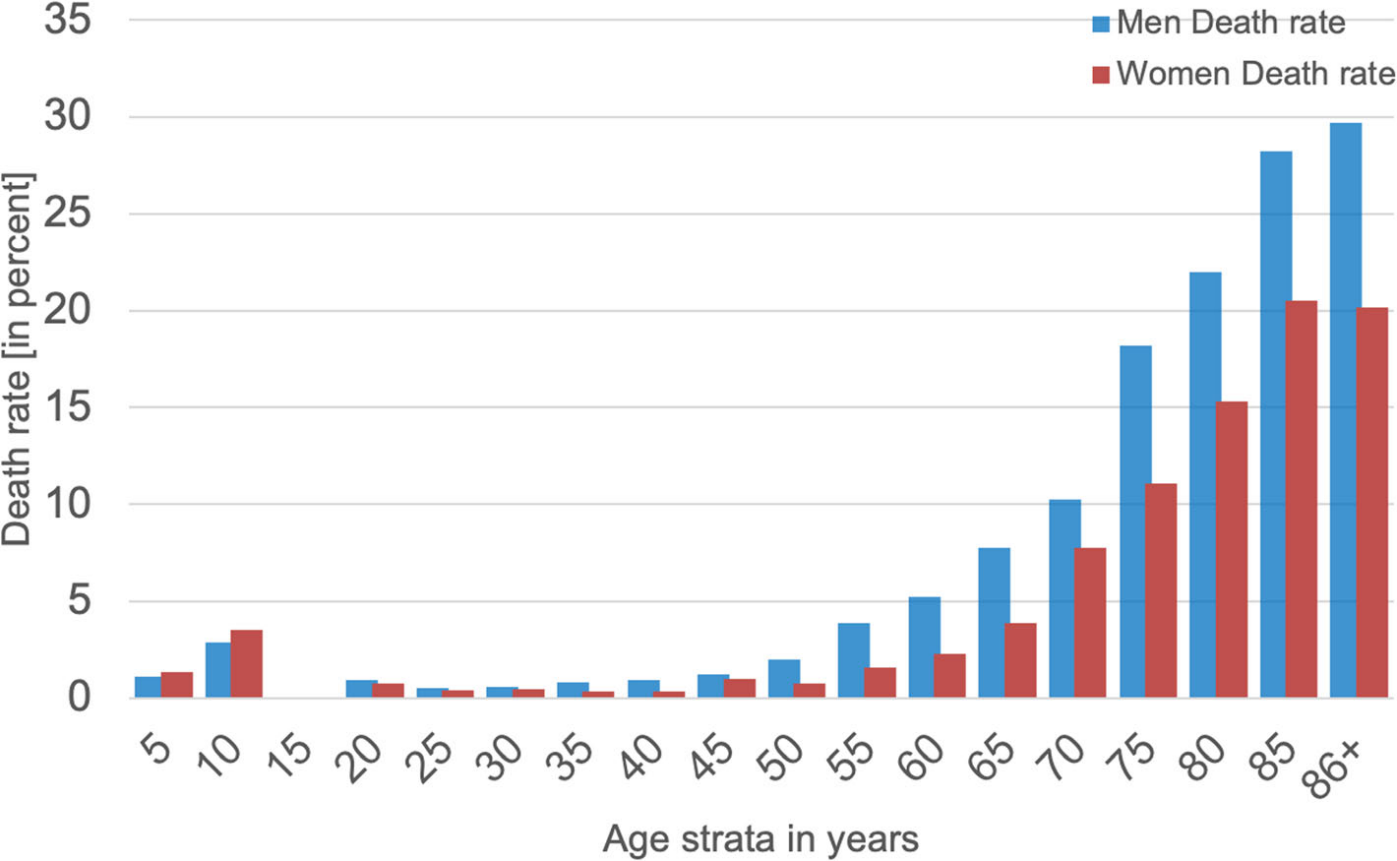
A histogram of death rates in percent for women (red) and men (blue) in 5-year age strata: cumulatively, men were more vulnerable to SARS-CoV-2 infection, with a death rate in some strata that is roughly + 50% higher than that in women

**Fig. 4:**
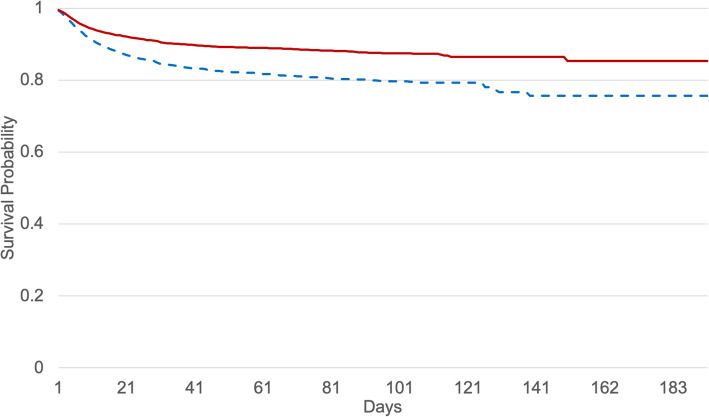
Kaplan-Meier survival curves for women [16,891] (red) and men [15,688] (blue, dashed), aged 50+ with SARS-CoV-2 infection

The results for the death rate show significant differences in women and men (*t* test, *p* < 0.01) (Fig. 3). Starting at age 45 years, the death rate increased for both men and women; men appear to be especially vulnerable to SARS-CoV-2 infection. The fatality rate is approximately + 50% higher in men vs. women (Fig. 3). For older men vs. women (those > 50 years of age), the calculated odds ratio (OR) was 1.68, 95% CI [1.55, 1.81]. Correspondingly, a higher risk of death was associated with men; the hazard ratio (HR) was 2.7 [1.42, 5.08] with a lower survival probability for men than women. At 200 days after diagnosis of SARS-CoV-2 infection, men were associated with a lower chance of survival than women (*p* < 0.0001), (Fig. 4).

### Results for the female sub-cohort: a link between exogenous estradiol and outcomes?

In order to assess the potential effects of exogenous female sex hormones in enhancing the survival likelihood for women with SARS-CoV-2 infection, we analyzed a sub-cohort of only women. A logistic regression analysis was performed for the combined outcome variable “death.” The outcome was calculated for *n* = 18,892 women, aged 15–49 years, who used oral contraceptives such as estradiol and ethinyl estradiol (defined as a “user” group) or without regular use of oral contraceptives (defined as “non-user”); we also included *n* = 16,891 peri- and post-menopausal women (50+), either with estradiol hormone usage (“user”) or without regular usage of estradiol hormone (“non-user”). Hormone use was identified via RxNorm codes 4083 (estradiol), 4124 (ethinyl estradiol), progestins VA:HS800, and systemic contraceptives VA:HS200. It was required that patients must have taken (or not taken) the drug within the last 1 year.

After matching the data of propensity scores for women aged 50+ with COVID-19, there was found to be a benefit for the estradiol hormone-user group vs. the non-user group, as regards an outcome of fatality. The OR calculated via logistic regression analysis for the combined outcome variable was 0.33 [0.18, 0.62] and the hazard ratio (HR) was 0.29 [0.11, 0.76] for the estradiol non-user vs. hormone-user group (Table 1). The average age across both groups was 64.2 years. The risk reduction for fatality from 6.6% (non-user) to 2.3% (user) was statistically significant (*p* < 0.0001).

**Table 1:** Cohort of estradiol user and non-user women aged 50+

A.Estradiol usage cohort (♀ > 50 years old)	Patients in cohort	Odds ratio [95% CI]	Hazard ratio [95% CI]
User	439	0.33 [0.18, 0.62]	0.29 [0.11, 076]
Non-user	16,278		

The data for women 15–49 years of age, who were either documented users or non-users of oral contraception (estradiol, ethinylestradiol), were analyzed by logistic regression models (Additional files 2, 3, 4, 5 and 6). The effect was smaller than in the peri- and post-menopausal group aged 50+. The OR calculated with logistic regression analysis for the combined outcome variable “death” was 1.0 [0.41, 2.4] for the non-user group vs. the oral contraception-user group (Table 2).

**Table 2:** Cohort of oral contraception user and non-user women 15–49 years of age

Women 15–49 years Oral contraception	Patients in cohort	Odds ratio [95% CI]	Hazard ratio [95% CI]
User	2078	1.0 [0.41–2.4]	0.28 [0.1–1.1]
Non-user	16,814		

### Survival probability

**Fig. 5:**
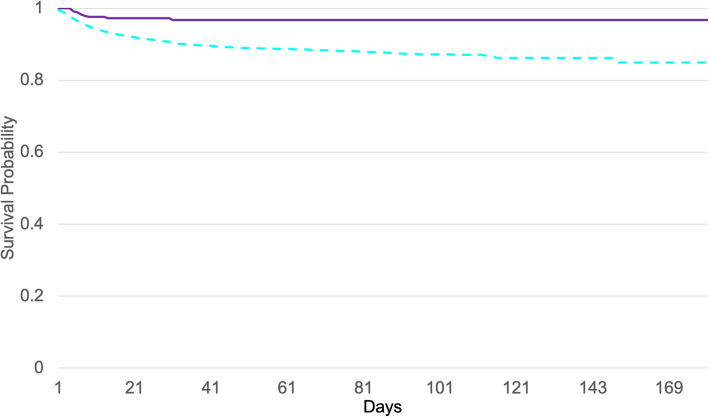
Kaplan-Meier survival curves: The survival probability of age 50+ women who were estradiol users (violet line) is shown, alongside non-users (blue dashed line)

Survival probabilities were calculated via Kaplan-Meier analyses, which revealed a significant difference (*p* < 0.0001) among women for the 50+ estradiol user group (*n* = 439) compared to the age-matched estradiol non-user group (*n* = 16,278). Considering the survival probability at 180 days after diagnosis of SARS-CoV-2 infection (index event), *n* = 10 women died in the estradiol user group and *n* = 1072 died in the non-user group. Peri- and post-menopausal women, aged 50+, benefitted from estradiol hormone use: the 180-day survival probability for this cohort was 96.7%, compared to 84.9% for the non-user group (Fig. 5 Additional files 7, 8, 9, 10 and 11).

## Discussion

This study focuses on the incidence and outcome of COVID-19 infections by considering an age- and sex-disaggregated data analysis. We identified a sex-specific distribution of COVID-19 incidence rates, with the highest frequencies being among premenopausal women in the 20–55-year age range. We also found a higher fatality rate of men compared to age-matched women, beginning at 50 years of age, and that estradiol hormone use reduced fatality rates for women in this 50+ age range.

The patterns of incidence rates of women and men with SARS-CoV-2 infection, measured using 5-year age strata, differs from the trends in the number of deaths following the same age strata (Fig. 3). This age-dependent, sex- and gender-based discrepancy might hint at different factors as contributing to the differential risks of infection and COVID-19 fatality among women versus men; note that these other factors may be either intrinsic (e.g., physiological/mechanistic) or extrinsic (e.g., smoking, lifestyle patterns or other circumstances) in nature. Gebhard et al. recently published the first sex-disaggregated data of confirmed COVID-19 cases and deaths, provided by Global Health 50/50 data tracker. Data from China and Europe were evaluated. Regardless of country-specific demographics, case fatality in men was higher than in women for all age groups and was more evident in middle age [17]. Similarly, Klein et al. reported in the USA a significant male-female difference in COVID-19 cases, hospitalizations, and deaths [18].

The occurrence of most cases of SARS-CoV-2 infection within the reproductive ages—i.e., before age ~ 50 (see Fig. 2 and the dip towards the right-hand side)—hints at an association with relatively higher estrogen levels. General features of the datasets used in our large international COVID-19 cohort, comprising *n* = 68,466 cases, are consistent with the public data for the incidence of coronavirus infection and the fatality rate for COVID-19 disease in Germany, as presented by the Robert-Koch Institute (RKI) website [19]. In our study, the incidence of coronavirus infection in young women is about + 15% higher than age-matched men; however, the fatality rate is about + 50% higher in men than women.

The data in our present study indicate that pre-menopausal women are disproportionately more infected with coronavirus than men in the same age brackets, but they do not become as seriously ill, as evidenced by lower fatality rates; we believe this to be an interesting observation for sex and gender medicine experts.

Sex- and gender-specific epidemiological differences, with pathophysiological bases, have been published in the recent past, pointing to potential mechanistic roles for the sex hormones. However, the X-chromosomal location of the ACE2- and the AT2-receptor genes—and the downregulation of the pleiotropic cytokine interleukin 6 (IL-6) by estrogens and its upregulation by androgens—should also be considered, along with other biochemical and physiological factors to which sex-correlated discrepancies might be attributed [20]. A central molecular component here is human ACE2, which is known to interact strongly with the SARS-CoV-2 viral spike protein [11]; together with the “transmembrane protease, serine 2” (TMPRSS2) enzyme, ACE2 plays an essential role in viral entry of SARS-CoV-2 into host cells [6].

Pre-menopausal women are more protected from cardiovascular, lung, and kidney diseases than their age-matched male peers. Recently, it was shown that the estrogen-mediated upregulation of the Mas-receptor contributes to the prevention of acute lung injury and affects endothelial barrier stabilization [21]. A relative protection of females over males has also been observed in other studies with experimental animal models of acute lung injury [22]; intriguingly, this protection was lost in ovariectomized mice, but restored upon estrogen replacement [23]. The 17β-estradiol molecule attenuates lung vascular permeability and edema [24], and estrogens reduce the pulmonary vasoconstriction during hypoxia by increasing levels of prostacyclin and nitric oxide (NO) [24].

The ACE2 and AT2R genes map to the X chromosome. In genetic terms, this sex-linkage would allow women to be heterozygous and differently assorted compared to men, who are hemizygous [25]. The second X chromosome is not inactivated in approximately 15% of genes and another 15% of genes vary between individuals or tissues in whether they are subject to, or escape from, inactivation [26]. This may account for some of the differences that are seen between men and women (sexual dimorphism) and could be a reason for higher expression levels of ACE2- and ATIIR proteins in women (i.e., a gene dosage effect).

Another pertinent mechanistic pathway, potentially triggered or modulated by the types and levels of sex hormones, could be immunological—namely, sex-specific differences in the immune response to SARS-CoV-2 infection. A recent study has linked higher mortality among men to a “cytokine storm,” which in turn closely relates to the severity of symptoms such as pulmonary edema, fibrosis, and other deleterious downstream effects associated with acute lung injury [27]. An individual’s immune response to viral infections can vary with fluctuations in sex hormone concentrations, including steroid hormones such as estrogens, progesterone, and testosterone. Each immune cell type is influenced by all three of these major sex hormones, albeit with different and partly opposing effects. From a biomedical perspective of sex, interleukin-6 (IL-6) is particularly interesting. IL-6 is a cytokine with both anti- and pro-inflammatory effects. It can be produced by almost all stromal and immune system cells (e.g., monocytes, lymphocytes, macrophages, endothelial cells, mast cells, dendritic cells), and it is believed to play a central role in precipitating the cytokine storms that, in turn, yield such severe symptoms from a SARS-CoV-2 infection [28]. Notably, the endogenous female sex hormone 17β-estradiol blocks the IL-6 cytokine pathway, while the male sex hormones (the androgens) increase IL-6 production [20]. We again employed the COVID-19 cohort to evaluate the impact of exogenous estradiol sex hormonal use, either for contraceptive purposes or for postmenopausal symptoms, on COVID-19 fatality.

Among post-menopausal women, we observed a significant difference in the rates of death between women with regular estradiol use (user group) and those without estradiol sex hormone intake (non-user group). To the best of our knowledge, this important finding—that the fatality risk for women > 50 years receiving estradiol therapy (user group) is reduced by more than 50% (OR 0.33, 95% CI [0.18, 0.62] and hazard ratio 0.29, 95% CI [0.11,0.76]) compared to the non-users group—is described here for the first time. Furthermore, our data analysis included distinct sub-groups of young women (15–49 years of age) with and without oral contraceptives. The dominant form of estrogen used for “the pill” is ethinylestradiol. The magnitude of the protective effect of the usage of oral contraception, compared to the non-users sub-population, is smaller among younger versus older women; this may be the case because endogenous estradiol levels are typically already higher in younger women than for post-menopausal women, thus drowning out any differences between user/not-user groups. Moreover, the level of exogenous hormone intake for purposes of contraception is generally less than that used for post-menopausal estradiol hormone therapy.

Note that some current trials are testing the effect of sex hormones (estrogen and testosterone) on COVID-19 outcomes. A brief, 7-day course of estradiol, delivered via a transdermal patch, could be a safe approach to reduce symptom severity in adult men and in older women, when administered prior to intubation (ClinicalTrials.gov; Identifier: NCT04359329). The time-course for estradiol treatment may need to be evaluated for its positive effects on lung protection, and whether it could be an effective therapeutic approach not only for women but also for men with COVID-19. This type of question cannot be answered solely by the data presented here, and instead addressing this question is a key topic for further research.

Based on the main findings of our present study, we believe there are no concerns for continuing the use of sex hormones that contain estradiol prior to SARS-CoV-2 infection. Even though it can be seen in the data that the risk of infection is higher in pre-menopausal women with higher endogenous estradiol levels, compared to either men of the same age strata or to post-menopausal women, the clinical course of COVID-19 disease, and the ultimate mortality rate, is lower in women with higher estradiol levels. Higher survival probabilities are particularly evident in post-menopausal women who are infected with SARS-CoV-2 and who regularly use exogenous estradiol (e.g., for postmenopausal complaints).

## Conclusions

Pre-menopausal women are at a relatively high risk for SARS-CoV-2 infection, but the survival probability in this below-50 age range is significantly higher in women than in men. A chief finding of this study is the strong positive effect of regular estradiol hormone therapy on the survival rates of post-menopausal women.

## Supplementary Information

**Additional file 1.** Definition of COVID-19.

**Additional file 2.** Query definition 15–49 years.

**Additional file 3.** Measures of Association Data Graph 15–49 years.

**Additional file 4.** Measures of Association Data Table 15–49 years.

**Additional file 5.** Kaplan-Meier Raw Data Graph 15–49 years.

**Additional file 6.** Kaplan-Meier Raw Data Table 15–49 years.

**Additional file 7.** Query definition > 50 years.

**Additional file 8.** Measures of Association Data Graph > 50 years.

**Additional file 9.** Measures of Association Data Table > 50 years.

**Additional file 10.** Kaplan-Meier Raw Data Graph > 50 years.

**Additional file 11.** Kaplan-Meier Raw Data Table > 50 years.

## Data Availability

The data that support the findings of this study are available from TriNetX. While restrictions apply to the availability of these data, which were used under license for the current study and are not publicly available, the data are available from the authors upon reasonable request and with the permission of TriNetX.

## Abbreviations

COVID-19: Coronavirus disease 2019
ACE: Angiotensin-converting enzyme
OR/HR/CI: Odds ratio/hazard ratio/confidence interval
SARS-CoV-2: Severe acute respiratory syndrome-coronavirus-2
AT2R: Angiotensin II receptor type 2
RAAS: Renin angiotensin aldosterone system
IL-6: Interleukin-6
Ang1-7: Angiotensin 1-7
EHR: Electronic health records
RWE: Real-world evidence
